# Effect of the polyphenol flavonoids fisetin and quercetin on the adipogenic differentiation of human mesenchymal stromal cells

**DOI:** 10.1042/BSR20240623

**Published:** 2024-10-23

**Authors:** Chanchao Lorthongpanich, Thanapon Charoenwongpaiboon, Praphasri Septham, Chuti Laowtammathron, Pimonwan Srisook, Pakpoom Kheolamai, Sirikul Manochantr, Surapol Issaragrisil

**Affiliations:** 1 Department of Medicine, Siriraj Center of Excellence for Stem Cell Research, Faculty of Medicine Siriraj Hospital, Mahidol University, Bangkok, Thailand; 2 Department of Chemistry, Faculty of Science, Silpakorn University, Nakhon Pathom 73000, Thailand; 3 Center of Excellence in Stem Cell Research and Innovation, Faculty of Medicine, Thammasat University, Pathumthani 12120, Thailand; 4 Division of Hematology, Department of Medicine, Faculty of Medicine Siriraj Hospital, Mahidol University, Bangkok, Thailand; 5 Bangkok Hematology Center, Wattanosoth Hospital, BDMS Center of Excellence for Cancer, Bangkok, Thailand

**Keywords:** adipogenesis, Fisetin, Flavonoid, mesenchymal stem cell, osteogenesis, Quercetin

## Abstract

Fisetin and quercetin, polyphenol flavonoids, have been shown to have a wide range of beneficial pharmacological effects including anti-inflammatory, antioxidative, and anti-cancer. Our previous work shows that fisetin also affects the specification of the adipogenic-osteogenic lineage of human mesenchymal stem cells (hMSCs) by modulating the Hippo-YAP signaling pathway. Although quercetin has a structure similar to that of fisetin, its effects on the functional properties of hMSCs have not yet been investigated. The objective of the present study is to determine the effects of quercetin on the various properties of hMSCs, including proliferation, migration, and differentiation capacity toward adipogenic and osteogenic lineages. The results show that while fisetin increases hMSC adipogenic differentiation, quercetin inhibited adipogenic differentiation of hMSCs. The inhibition is mediated, at least in part, by the activation of hippo signaling and up-regulation of miR-27b, which inhibits the expression of genes involved in all critical steps of lipid droplet biogenesis, resulting in a decrease in the number of lipid droplets in hMSCs. It is possible that the lack of hydroxylation of the 5 position on the A ring of quercetin could be responsible for its different effect on the adipogenic-osteogenic lineage specification of hMSCs compared with fisetin. Molecular docking and molecular dynamics simulation suggested that fisetin and quercetin possibly bind to serine / threonine protein kinases 4 (STK4/MST1), which is an upstream kinase responsible for LATS phosphorylation. Taken together, our results demonstrate more insight into the mechanism underlying the role of flavonoid fisetin and quercetin in the regulation of adipogenesis.

## Introduction

Human mesenchymal stromal cells (hMSCs) are widely recognized as a promising source for stem cell therapy and tissue engineering due to their ability to self-renew and differentiate into various cell types. As a common progenitor, MSCs have to maintain a delicate balance for their differentiation commitment, especially for differentiation to fat and bone [1,2]. Multiple physical and chemical signals have been shown to affect hMSC functions by modulating the activity of various signaling pathways and transcriptional regulators. It has been shown that bone and fat differentiation of MSCs is a competing and reciprocal process. Bone induction factors, such as RUNX family transcription factor 2 (Runx2), inhibit adipogenesis, while the peroxisome proliferator-activated receptor γ (PPARγ) stimulated adipogenesis and inhibited osteogenesis [3]. Several factors have been suggested as critical factors that affect adipo-osteogenic decision, including flavonoid fisetin [2].

Flavonoids are polyphenolic chemicals that are abundantly found in various fruits and vegetables. All flavonoids have a typical structure of C6-C3-C6, where two benzene rings, each composed of six carbon atoms, are linked by a central ring consisting of three carbon atoms [4]. Numerous studies have shown that flavonoids have a wide range of beneficial pharmacological effects including anti-inflammatory, antioxidative, and anti-cancer. Furthermore, studies also show that flavonoids affect various biological properties of hMSCs, especially their osteogenic differentiation [2,5]. For example, quercetin has been shown to promote the proliferation and osteogenic differentiation of bone marrow-derived MSCs through a pathway mediated by estrogen receptors [6]. Naringin enhances osteogenic differentiation by activating the AMPK/AKT signaling pathway [7,8]. Glabridin induces osteogenic differentiation of MSCs by increasing the expression of the *OCT4* gene [9], while catechin stimulates osteogenesis in hMSCs by increasing PP2A activity [10]. Our previous study shows that fisetin inhibits osteogenic differentiation of hMSCs by activating the Hippo-YAP signaling pathway and preventing the formation of the TEAD-YAP complex [2,11]. These results clearly demonstrated the effect of flavonoids on osteogenesis. However, the effect of flavonoids on other lineages of MSC differentiation, such as adipogenesis, is not well studied.

**Figure 1:**
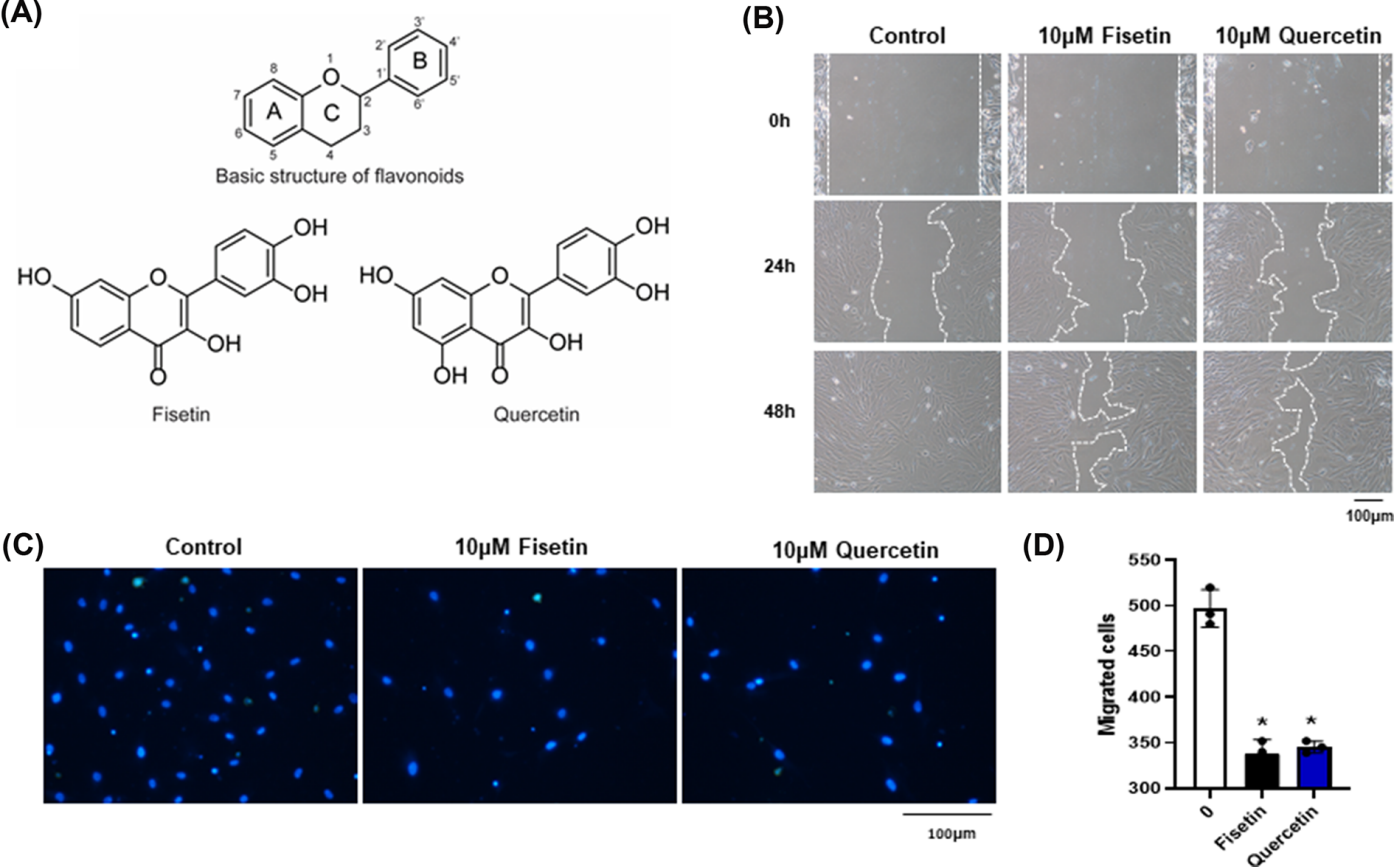
Basic chemical structure of flavonoids and their effect on hMSC migration Chemical structure of fisetin and quercetin (**A**). A scratch of human MSCs was made using a P1000 pipette tip before culturing in the presence of 10 µM fisetin or 10 µM quercetin. The overall migration was observed every 24 h. (**B**). Representative pictures of cells on the bottom side of the inserted chamber after incubation with fisetin and quercetin for 6 h and stained with Hoechst 33342 in the Transwell migration assay (**C**). The number of migrated cells was counted and reported. Data are presented as mean ± standard deviation (SD) of three independent experiments. *p < 0.01 (**D**).

Among several flavonoids, quercetin is a flavonoid that has a structure very similar to that of fisetin, except that quercetin has an additional 5-hydroxyl group on the A ring (Figure 1A). Although structurally similar, previous studies have shown that fisetin and quercetin have a distinctive effect on several biological aspects, such as differences in antioxidant [12–16] and anti-cancer activities [13]. These studies suggest that the slight differences in chemical structure could be responsible for the differences in the biological activity of flavonoids. Although the effects of fisetin on the specification of osteogenic lineage [2] have been reported, its function on adipogenesis is not well understood. Therefore, in the present study, we aim to determine the effects of fisetin and quercetin on the functional properties of hMSCs focusing on the differentiation toward osteo and adipogenic lineages, which is a competing and reciprocal process. The results obtained from the present study could provide a better understanding of the underlying mechanism and suggest an association between fisetin and quercetin as a dietary supplement that regulates adipogenesis.

## Materials and methods

### Isolation, culture, and characterization of human mesenchymal stromal cells (hMSCs)

The placenta of healthy newborns was collected after receiving written informed consent from their mothers. Chorionic tissue was isolated and washed in phosphate buffer saline (PBS), cut into small pieces, before being treated with 0.25% Trypsin-EDTA (GIBCO™; Invitrogen Corporation, Carlsbad, CA, USA) for 30 min at 37°C. Cells were harvested and cultured in Dulbecco's modified eagle medium (DMEM) supplemented with 10% (v/v) fetal bovine serum (FBS) and seeded in tissue culture vessels (Corning Incorporated, Corning, NY, USA). The cells were cultured at 37°C in a humidified atmosphere containing 5% CO_2_ in air. The culture medium was changed every two days. Cells were subjected to immunophenotypic characterization as previously described by Lorthongpanich et al. to examine the presence of specific surface molecules for MSC, including CD73, CD90, and CD105, and the absence of the hematopoietic surface markers CD34 and CD45 [2]. After incubation with the appropriate secondary antibody, cells were washed with PBS and fixed with 1% paraformaldehyde (PFA) (w / v) before analyzed by flow cytometer (FACSCantoTM or FACSCalibur TM analyzer; BD Biosciences, San Jose, CA, USA). All antibodies used in this assay were diluted to 1:100 dilutions in PBS.

### Preparation of fisetin and quercetin

Fisetin and Quercetin were purchased from Sigma-Aldrich Corporation (St. Louis, MO, USA) and dissolved in dimethylsulfoxide (DMSO) (Sigma-Aldrich) to obtain a stock solution of 10 mM. The stock solution was ultimately diluted in culture medium to obtain the desired concentration. The control group was treated with DMSO at a final concentration of 0.1% to eliminate the effect of the reagent. Both reagents were diluted to different concentrations and subjected to toxicity testing using the methylthiazol tetrazolium (MTT) assay. The absorbance at the wavelength of 570 nm was measured using a microplate reader (Synergy H1, BioTek Instruments, Inc., Winooski, VT, USA). The concentration of fisetin and quercetin that alters YAP activity but still shows a survival rate of more than 80% will be selected for further experiments (Supplementary Figure 1).

### Osteogenic differentiation and mineralization assay

The osteogenesis and mineralization assays were performed as previously described by Lorthongpanich et al. [11]. Briefly, MSCs were cultured in high glucose DMEM supplemented with 10% (v/v) fetal calf serum until their density reached 90% confluence. At this stage, DMEM was replaced with NH OsteoDiff® medium (Miltenyi Biotec, Bergisch Gladbach, Germany) to induce osteogenic differentiation of hMSCs according to the manufacturer's instructions. In the treatment groups, NH OsteoDiff® medium was supplemented with various concentrations of fisetin or quercetin, ranging from 0 to 10 µM. hMSCs cultured in NH OsteoDiff® medium without fisetin and quercetin supplementation serve as an untreated control. The medium was replaced every 3 d for the entire culture period. Calcium deposition was determined by an alizarin red S staining assay as described previously. Briefly, differentiated MSCs were fixed with 4% (w/v) PFA, washed twice with deionized water, and stained with 40 mM Alizarin Red S (Sigma-Aldrich, USA) for 20 min at room temperature (RT). The level of calcium deposition was quantified by the amount of Alizarin red S retained by the cells. The quantification of Alizarin Red S was performed as previously described by Lorthongpanich et al. [11].

### Adipogenic differentiation

Adipogenic differentiation was performed as previously described by Klincumhom et al. [17]. Briefly, MSCs at a density of 5 × 10^4^ cells were seeded in 1 in 6-well plates (Corning) and cultured for 21 d in an adipogenic differentiation medium. This consisted of high glucose DMEM (Gibco) supplemented with 1 μM dexamethasone (Sigma-Aldrich), 0.5 μM isobutylmethylxanthine (Sigma-Aldrich), 5 μg/ml insulin, and 50 μM indomethacin (Sigma-Aldrich). The differentiation efficiency was determined by qRT-PCR for the expression of adipogenic-specific genes. Cells were assessed for intracellular lipid accumulation using 0.5% (w / v) with Oil Red O (Sigma Aldrich) for 30 min at RT. The dye was eluted with 100% isopropanol by incubating cells with isopropanol for 10 min at RT. Solution aliquots of 200 μl/well were transferred to a 96 well plate to determine the absorbance was at 510 nm using a spectrophotometer (BioTek Instruments Inc., Winooski, VT, USA).

### Proliferation assay

The MTT assay was used to determine the cytotoxicity of fisetin and quercetin. The MSCs at a density of 5 × 10^3^ cells were seeded in each well of the 96-well plate and incubated at 37°C, 5% CO_2_ overnight. MSCs were treated with different concentrations of fisetin or quercetin ranging from 0–100 μg/ml. At 72 h of incubation, MTT solution (final concentration 0.5 mg/ml) was added to each well and incubated at 37°C for 3 h. After the medium was removed from the plate, 100µl of DMSO was added to each well to dissolve the purple formazan crystals. A Bio Tek Quant universal microplate spectrophotometer (Synergy H1, BioTek Instruments, Inc., Winooski, VT, USA) was used to measure cells at 570 nm and a reference wavelength of 630 nm. Cell viability was measured as a percentage ratio and compared with control cells.

### Scratch wound migration assay

MSCs at a concentration of 8 × 10^4^ cells/well were seeded in 24-well plates and cultured overnight. At 80% to 90% confluence, cells were wound by scratching the surface of the culture well with a sterile 1000 ml pipette tip. Cells were washed with 1xPBS to remove detached cells and other cellular debris before adding Fisetin and Quercetin at different concentrations (0 and 10 μM) and incubated at 37°C, 5% CO_2_ for 48 h. Inverted microscopy was used to capture images of the closing wound at 0, 24, and 48 h.

### Transwell migration assay

The MSCs were treated with 0 or 10 µM fisetin or quercetin for 24 h before seeding in the insert chamber of an 8 µm pore size transwell (Corning, NY, USA) filled with DMEM supplemented with 2% (v/v) FBS, 100 U/ml penicillin and 100 µg/ml streptomycin. The lower chamber contained 600 µL of DMEM medium supplemented with 20% FBS. Migration was observed 6 h after culture at 37°C, 5% CO_2_ in air. Migrant cells were fixed with 4% (x/v) paraformaldehyde for 20 min before washed with PBS and stained with Hoechst 33342 (Thermo Scientific, MA, USA). The stained cells were then imaged and counted. Three independent experiments were performed. Data are presented as mean ± standard deviation (SD).

### Transfection of the miR-27b inhibitor

A total of 2.5 × 10^5^ CH-MSC cells were seeded in a T25 tissue culture flask 1 d before the study on the role of miR-27b in CH-MSC cells. A 100 nM miR-27b inhibitor (has-miR-27b-3p, miRbase accession #MIMAT0000419 purchased from Invitrogen by Thermo Fisher Scientific, MA, USA) was transfected into cells using the Lipofectamine^TM^ 3000 transfection kit (Invitrogen by Thermo Fisher Scientific, MA, USA). Transfected cells were harvested 72 h after transfection for further experiment.

### Western blot analysis

The treated MSCs were lysed in RIPA buffer supplemented with protease inhibitors. Total proteins were loaded and separated on a 7.5% SDS-PAGE gel. After electrophoresis, the proteins were transferred to the PVDF membrane (Merck Millipore, MA, USA), blocked in 5% (w/v) skim milk, and incubated with the primary antibodies at 4°C overnight. The primary antibodies used were as follows: anti-YAP, anti-phosphorylated YAP, anti-LATS1, anti-phosphorylated LATS(T1079) (Cell Signaling Technology, MA, USA) diluted 1:1,000 and anti-β-actin peroxidase (ACTB; Sigma-Aldrich, MO, USA) diluted 1:10,000. The membrane was washed with Tris-buffered saline/Tween 3 times and incubated with the secondary antibodies at RT for 2 h. Signals were visualized using a Lumina crescendo western horseradish peroxidase substrate (Millipore, MO, USA). The relative intensity of the bands was measured using ImageJ software.

### Real-time quantitative reverse transcription polymerase chain reaction (real-time qRT-PCR)

RNA was isolated from experimental cells, and reverse transcription was performed using a high-capacity cDNA reverse transcription kit (Applied Biosystems, CA, USA), qRT-PCR was performed according to the manufacturer's instructions. Real-time qRT-PCR was performed using Real-Time PCR Master Mix (Applied Biosystems) using a CFX384 Real-Time PCR System (Bio-Rad Laboratories, CA, USA). The primers used in the present study are listed in Supplementary Tables S1 and S2.

### Statistical analysis

Results are presented as mean ± SD of three independent experiments. The Mann–Whitney U test was used to compare non-parametric variations between groups. A *p*-value of <0.05 was considered statistically significant. Data were analyzed using GraphPad Prism software version 8.0 for Windows (GraphPad Software, CA, USA).

### Computational analysis

Homology model of serine/threonine-protein kinase 4 (STK4) was built by Swissmodel server [18,19]. The protonation state of the protein was determined at pH 7.4 using a H++ server. The structures of fisetin and quercetin were downloaded from PubChem [19]. Autodock vina was used for molecular docking [20]. Molecular dynamics simulation (MD) was performed using AMBER20 as previously described. The MMPBSA.py module (AMBER) [21] was used to calculate the binding affinity between STK4 and compounds based on the MM/GBSA method [22].

## Results

### Quercetin and fisetin inhibit the migration of hMSC

Fisetin has previously been shown to inhibit the proliferation and migration of hMSCs [2]. To determine whether slight differences in the molecular structure between fisetin and quercetin cause a difference in the effects of these two flavonoids on hMSC migration, we performed a scratch wound healing assay to determine the migration capacity of hMSCs after treatment with fisetin and quercetin. The results showed that 10 μM fisetin and 10 μM quercetin inhibited hMSC migration compared with the untreated control (Figure 1B). Consistent with the scratch wound assay, the transwell migration assay also showed that 10 μM fisetin and 10 μM quercetin significantly reduced the number of migrated hMSCs compared with the untreated control (340 ± 12 cells vs. 491 ± 35 cells, *P < 0.05 and 345 ± 9 cells vs. 491 ± 35 cells, *P < 0.05, respectively) (Figure 1C,D). Altogether, these results suggest that both fisetin and quercetin inhibit the migration of hMSC.

### Quercetin and fisetin have different effects on the adipogenic-osteogenic lineage specification of hMSCs

**Figure 2:**
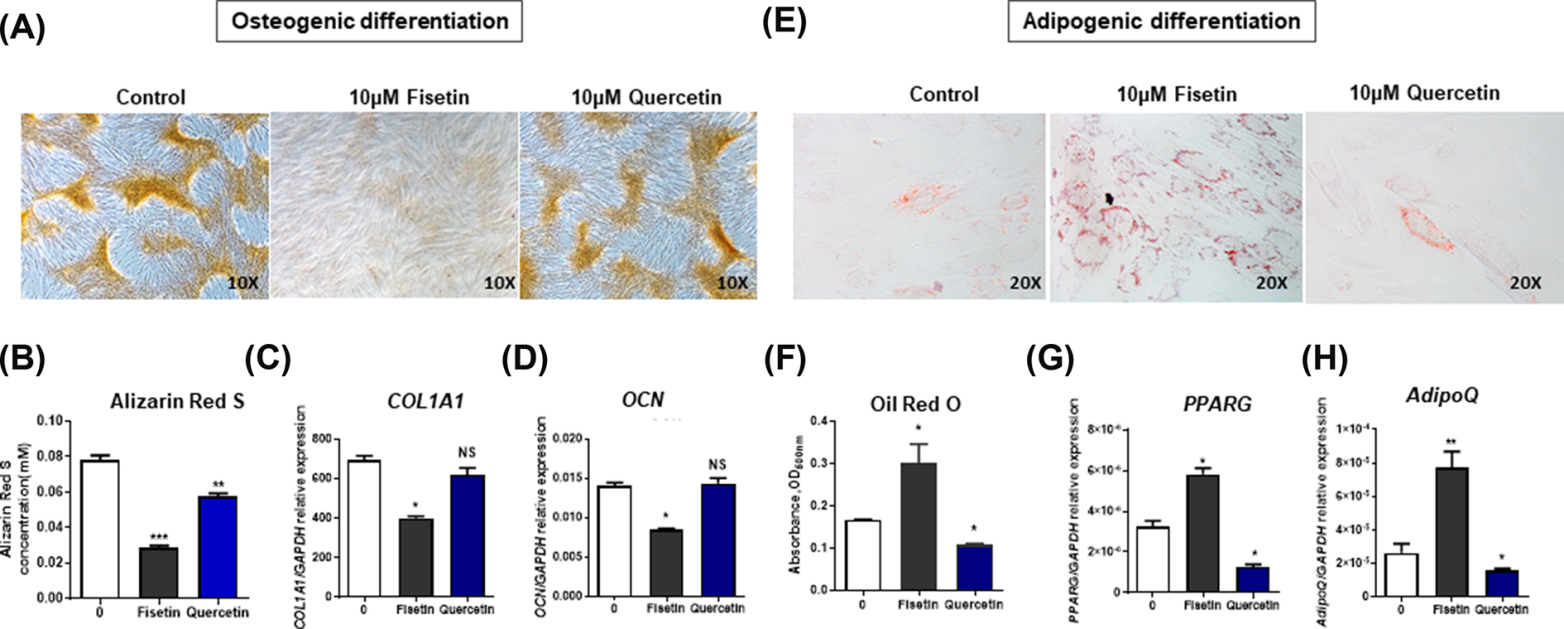
Osteogenic and adipogenic differentiation of human MSCs after treatment with fisetin and quercetin Alizarin red (**A**) and osteogenic gene expression (**B–D**) in hMSC-derived osteoblasts. Oil red O staining (**E**) and adipogenic gene expression in hMSC-derived adipogenic cells after treatment with fisetin and quercetin. Data are presented as mean ± SD of three independent experiments. *p < 0.05, **p < 0.01, ***p < 0.001, ns = non-significant difference.

To determine whether fisetin and quercetin affect the differentiation capacity of hMSCs, the hMSCs were subjected to osteogenic and adipogenic differentiation with fisetin or quercetin supplementation throughout the differentiation time course. Result showed that 10 μM fisetin inhibited osteogenic differentiation of hMSCs, as demonstrated by the significant reduction in calcium mineralization determined by alizarin red S staining (Figure 2A,B), and expression levels of the osteogenic genes, *COL1A1* and *OCN* were also down-regulated in the fisetin treated group (Figure 2C,D). However, 10 μM quercetin only slightly reduced the level of alizarin red S staining (Figure 2A,B) and did not affect the expression levels of *COL1A1* and the *OCN* genes (Figure 2C,D). These results demonstrate the distinctive effect of fisetin and quercetin on hMSC osteogenic differentiation.

In contrast with the inhibitory effect of fisetin on the osteogenic differentiation, we found that fisetin enhanced adipogenic differentiation of hMSCs, as demonstrated by the significant increase in cytoplasmic lipid droplet accumulation determined by oil-red O staining (Figure 2E,F), and up-regulation of the expression levels of adipogenic genes, *PPARγ* and *ADIPOQ* compared with untreated control (Figure 2G,H) which suggests function of fisetin in regulating the adipo-osteogenic balance of hMSCs. Interestingly, 10 μM quercetin had the opposite effect on hMSCs by suppressing their adipogenic differentiation, as determined by the significant reduction in oil-red O staining (Figure 2E,F), and the down-regulation of the expression levels of *PPARγ* and *ADIPOQ* genes (Figure 2G,H) compared with the non-treated control. This result suggests that while fisetin increases adipogenic differentiation of hMSCs, quercetin has the opposite effect by suppressing adipogenic differentiation of these cells. Furthermore, while fisetin strongly suppresses osteogenic differentiation of hMSCs, quercetin only minimally affects this property.

### Effect of quercetin on the biogenesis of lipid droplets in hMSC-derived adipocytes

**Figure 3:**
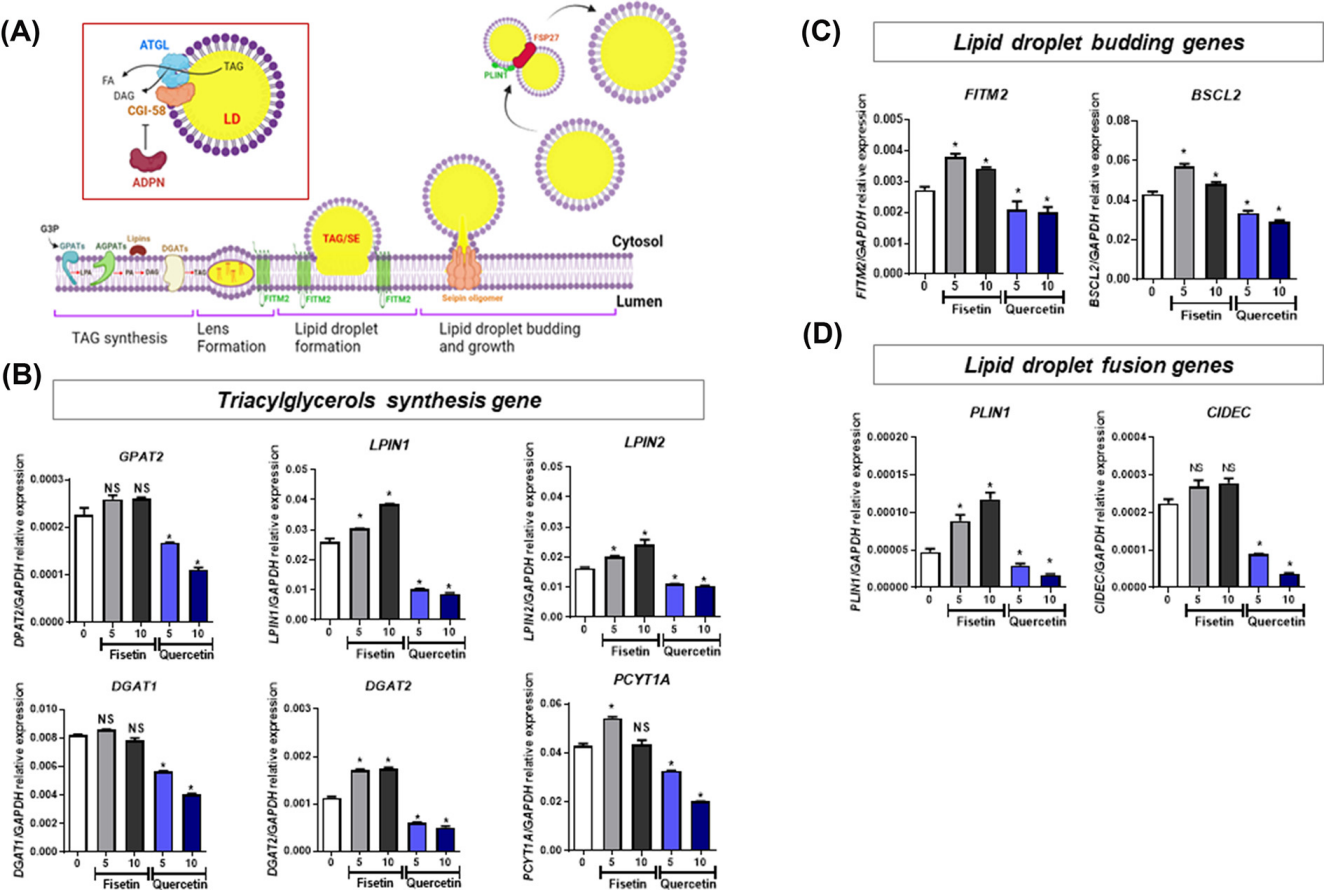
Expression of genes in lipid biogenesis after treatment with fisetin and quercetin The schematic diagram shows the biogenesis of lipid droplets and the relevant genes at each step of the process (**A**), the transcriptional analysis for the expression of genes involving in triglyceride synthesis (**B**), lipid droplet budding (**C**) and lipid droplet fusion (**D**). Data are presented as mean ± SD of three independent experiments. *p < 0.05, ns = non-significant difference.

To better understand the effect of fisetin and quercetin on human lipogenesis, genes involved in the various steps of lipid droplet biogenesis (Figure 3A, modified from [23]) were determined in hMSCs that were subjected to adipogenic differentiation in the presence of fisetin or quercetin for 21 d. The results show that fisetin enhances the expression levels of *PPARγ* and *ADIPOQ* genes (Figure 2G,H), and significantly increased the expression levels of the genes involved in triglyceride synthesis (*LPIN1, LPIN2, DGAT1, DGAT2* and *PCYT1A*) (Figure 3B), lipid droplet budding (*FITM2* and *BSCL2*) (Figure 3C), and lipid droplet fusion (*PLIN1* and *CIDEC*) (Figure 3D). On the other hand, quercetin suppresses the expression levels of *PPARG* and *ADIPOQ* genes (Figure 2G,H), and significantly decreased the expression levels of genes involved in triglyceride synthesis (*LPIN1, LPIN2* and *DGAT2*), the budding of lipid droplets (*FITM2* and *BSCL2*), and the fusion of lipid droplets (*PLIN1* and *CIDEC*) (Figure 3B-D). These results confirm that while fisetin induces adipogenic differentiation of hMSCs, possibly by up-regulating the expression levels of several genes involved in all critical steps of lipid droplet biogenesis, quercetin suppresses adipogenic differentiation of hMSCs by down-regulating the expression levels of these adipogenic genes.

### Quercetin induces the expression of miR-27b, resulting in inhibition of adipogenesis

**Figure 4:**
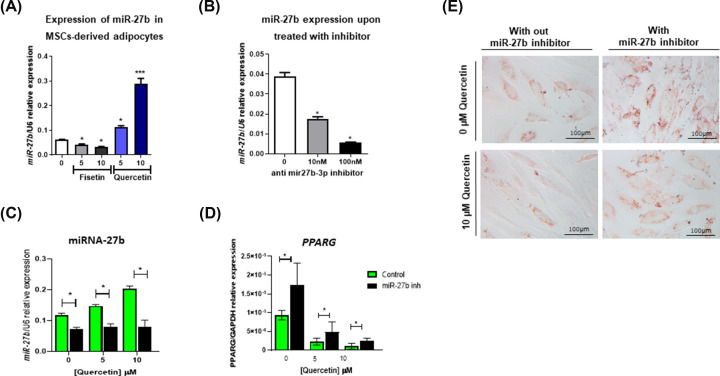
Expression and function of miR-27b after treatment with fisetin and quercetin Expression of miR-27b in hMSC-derived adipocytes after treatment with fisetin or quercetin (**A**). Expression level of miR-27b before and after treatment with the miR-27b inhibitor (**B**). Expression level of miRNA-27b on day 21 (**C**) and *PPARG* (**D**) after treatment with miR-27b inhibitor. Representative pictures of oil-red O staining in hMSC-derived adipocytes after treatment with quercetin and/or miR-27b inhibitor (**E**). Data are presented as mean ± SD of three independent experiments. *p < 0.05, ***p < 0.001.

To investigate the mechanism that mediates the effects of quercetin, we determined the expression levels of miR-27b, which have been shown to play an important role in the regulation of adipogenic differentiation of human adipogenic stem cells, after quercetin treatment. The result showed that quercetin, which inhibits adipogenic differentiation of hMSCs, significantly increases the level of miR-27b expression in a dose-dependent manner, while fisetin, which increases adipogenic differentiation, reduced the expression of this miRNA (Figure 4A).

To further determine the role of miR-27b in mediating the effect of quercetin, a miR-27b inhibitor (anti- mir27b-3p) was used to inhibit miR-27b expression during adipogenic differentiation of quercetin-treated hMSCs. The anti mir27b-3p successfully reduced the expression level of miR-27b in a dose-dependent manner (Figure 4B) and the 100nM concentration was used in further experiments. As expected, 100nM anti-mir27b-3p significantly reduced the expression level of miR-27b in hMSCs treated with 5 and 10 μM quercetin (Figure 4C). Consistent with this result, 100nM anti-mir27b-3p also significantly reduced the suppressive effect of quercetin on adipogenic differentiation of hMSCs, since it significantly increased the level of *PPARG* gene expression (Figure 4D) and the level of oil red o staining (Figure 4E) in quercetin-treated hMSCs. Altogether, these results suggest that quercetin inhibits adipogenic differentiation of hMSCs, at least in part, by increasing miR-27b expression.

### Effect of fisetin and quercetin on the activation of the Hippo-YAP signaling pathway

The Hippo-YAP signaling pathway has been shown to be an important regulator of adipo-osteogenic differentiation in hMSCs [11]. The activation of Hippo signaling pathway has been shown to induce adipogenic differentiation in hMSCs. Therefore, we hypothesized that quercetin, which inhibits hMSC adipogenic differentiation, could inhibit the Hippo-YAP signaling pathway in hMSCs.

**Figure 5:**
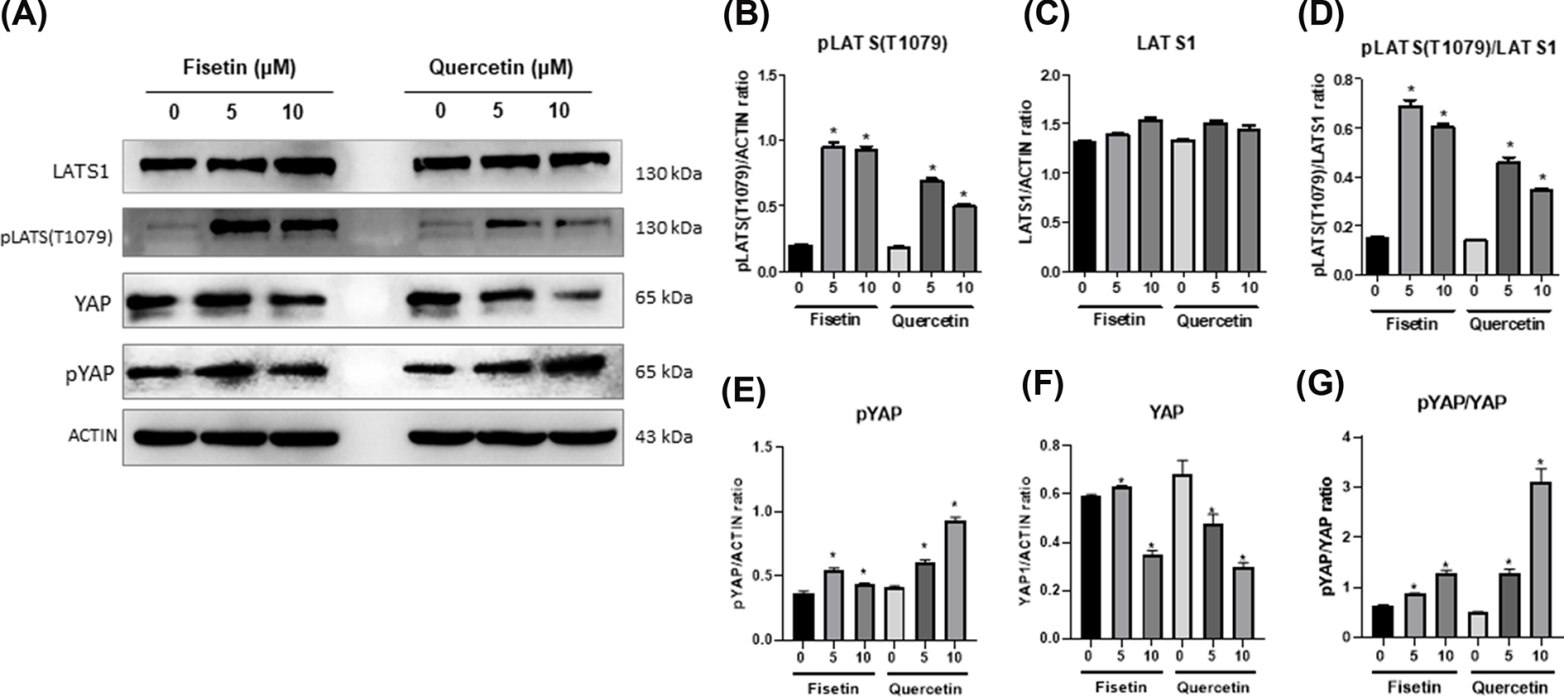
Effect of fisetin and quercetin on the Hippo-YAP signaling pathway Western blot analysis showed protein expression levels of LATS1, phosphorylated (p)-LATS, YAP and phosphorylated (p) -YAP after treatment with fisetin and quercetin (**A**). The intensity of the band of pLATS (**B**), LATS1 (**C**), and the pLATS/LATS1 ratio (**D**) after treatment with fisetin or quercetin. Band intensity of pYAP (**E**), YAP (**F**), and pYAP/YAP ratio (**G**) after treatment with fisetin or quercetin. Data are presented as mean ± SD of three independent experiments. *p < 0.05.

To determine whether the Hippo pathway is active, the phosphorylation state of LATS kinase can be used as an indicator. In the active pathway, LATS kinases will be phosphorylated by serine / threonine protein kinases 3/4 (STK3/4) to activate LATS. The active LATS (pLATS) subsequently phosphorylates and inhibits YAP activity, resulting in a reduction in YAP target gene expression [24]. As shown in our study, both fisetin and quercetin could activate a Hippo core kinase LATS1 in hMSCs, as there is an increasing level of pLATS when treated with fisetin and quercetin. However, fisetin activates the Hippo pathway at a higher level than quercetin (Figure 5A), as determined by the higher levels of phosphorylated LATS (pLATS), the pLATS/LATS ratio (Figure 5B-D), while quercetin shows a higher level of inactive YAP (pYAP) than fisetin (Figure 5E-G). These results suggest that fisetin actively activates the Hippo pathway than quercetin.

**Figure 6:**
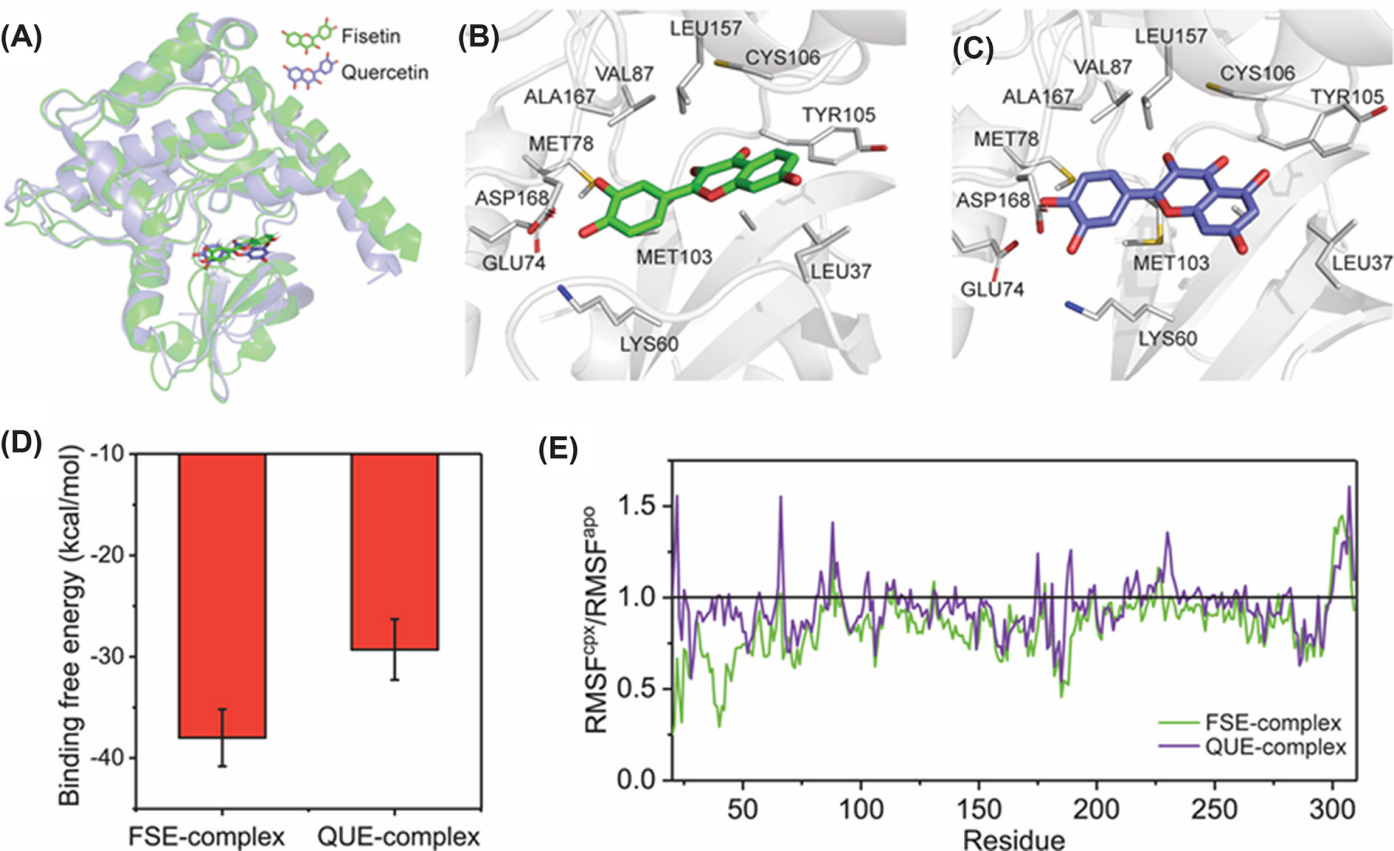
The 3D interaction and binding ability of fisetin and quercetin to the Hippo core kinase STK4 A molecular docking study of fisetin and quercetin in serine/threonine-protein kinases 4 (STK4/MST1) was performed (**A–C**). Blind docking to reveal the ability of fisetin and quercetin to bind to the ATP binding site of STK4 (**D**). The stability of STK4 in complex with fisetin and quercetin was evaluated using RMSF analysis (**E**).

A molecular docking study reveals that fisetin and quercetin can bind to the ATP binding site of STK4, which is an upstream core kinase of the Hippo signaling pathway responsible for LATS phosphorylation (Figure 6A–C). Fisetin exhibits a higher affinity for STK4 than quercetin, possibly due to repulsion between the hydroxyl group at C-5 of quercetin and a non-polar amino acid sidechain (Figure 6D). The binding of small molecules to the protein usually increases protein stability. RMSF analysis indicates that the stability of the STK4 protein increased after binding to fisetin compared with quercetin (Figure 6E), as indicated by the RMSF^apo^/RMSF^cpx^ ratio being less than 1, which could lead to an increase in LATS phosphorylation in fisetin than in the quercetin treated group as observed in Figure 5A.

Altogether, these results demonstrated that both fisetin and quercetin could act on the Hippo signaling pathway, but on a different protein target. Fisetin inhibits Hippo signaling activity by activating Hippo core kinase LATS1, resulting in an increase in inactive YAP (pYAP) and a reduction in active YAP. In contrast, quercetin barely activates LATS kinase, but rather suppresses YAP activity by increasing the level of inactive YAP (pYAP), resulting in a reduction in Hippo signaling pathway.

## Discussion

Quercetin is another flavonoid that has a structure closely similar to that of fisetin. However, several previous studies have shown that fisetin and quercetin have different antioxidant [12–16] and anti-cancer activities [13]. These results suggest that each flavonoid, despite having a similar molecular structure, could have different effects on the functional properties of hMSCs.

The present study reveals that although quercetin and fisetin show a similar effect on hMSC migration, they have different effects on the specification of the adipogenic-osteogenic lineage of hMSCs. Although fisetin strongly suppresses osteogenic differentiation of hMSCs, quercetin only minimally affects this property. It should be noted that our quercetin result, while consistent with several previous reports [25–28], still contradicts the result of Casado-Diaz et al. which shows that 10 µM quercetin inhibited osteogenic differentiation [25]. The contradiction could be due to the difference in cell types (bone marrow vs. placenta-derived MSCs) and the differentiation protocol used in these studies. Clarification of these differences could be further explored to determine the additional factor(s) affecting hMSC differentiation after quercetin treatment. Furthermore, while fisetin increases adipogenic differentiation by up-regulating the expression levels of several genes involved in all critical steps of lipid droplet biogenesis, quercetin suppresses the adipogenic differentiation of hMSCs by down-regulating the expression levels of these adipogenic genes. It is possible that the lack of hydroxylation of the 5 position on the A ring of quercetin could be responsible for its different effect on the adipogenic-osteogenic lineage specification of hMSCs compared with fisetin.

One of the less known and recently discovered is the ability of quercetin to modulate miRNA expression. Recent studies showed a strong impact of quercetin on various miRNA expressions, such as miR-146a in human breast cancer, miR-21 in prostate cancer cells [26], and miR-26b and miR-27b in pulmonary fibrosis [27]. Our results suggest that the inhibitory effect of quercetin on the adipogenic differentiation of hMSCs was mediated, at least in part, by increasing miR-27b expression. This is consistent with previous studies showing that miR-27b is negatively regulated during adipogenic differentiation of human multipotent adipose stem cells (hMADS) [28,29], and overexpression of miR-27b reduced the expression of adipogenic genes, *PPARγ* and *C/EBPα*, and inhibited the accumulation of lipid droplets in hMADS during its maturation stage.

In addition to its effect on increasing miR-27b expression, our results also show that quercetin could inhibit adipogenic differentiation of hMSCs by activating the Hippo signaling pathway, resulting in high levels of pLATS and pYAP proteins in these cells. This is consistent with previous studies showing that activation of Hippo signaling pathway increases adipogenic differentiation in hMSCs [2,30]. In addition, our western blot result shows that the level of pYAP is higher in quercetin compared with those of fisetin-treated cells. This result implied that quercetin might inhibit proteasome activity in hMSCs similar to what have been reported in cancer cells when treated with quercetin or other flavonoids with natural potent proteasome inhibitors, such as luteolin or apigenin [31,32]. However, more studies are needed to understand why this inhibitory effect happened only in p-YAP but not in p-LATS.

Although some evidence suggests that miR-27a and miR-27b might affect the activity of the Hippo signaling pathway, their roles have not yet been fully elucidated. Bioinformatic analysis and the luciferase reporter assay show that YAP, an important effector protein of the Hippo signaling pathway, is a direct target of miR-27 [33]. This is consistent with studies in models of cardiomyopathy, breast cancer, and oral squamous cell carcinoma showing that miR-27 inhibits YAP activity through a post-transcriptional silencing mechanism [34,35]. Furthermore, Chen et al. also found that Lats2 kinase, an upstream component of the Hippo signaling pathway, regulates miR-27 expression in liver cells [36]. Based on this evidence, it is possible that quercetin could inhibit human adipogenesis, at least in parts, by inhibiting the expression of YAP and PPARγ, a master regulator of adipogenesis, by increasing miR-27b expression.

## Conclusions

Although fisetin and quercetin are structurally similar, they have an opposite effect on the adipogenic differentiation of hMSCs. While fisetin increases hMSC adipogenic differentiation by up-regulating the expression levels of several genes involved in all critical steps of lipid droplet biogenesis, quercetin suppresses the adipogenic differentiation of hMSCs by down-regulating the expression levels of these adipogenic genes. The negative effect of quercetin on hMSC adipogenic differentiation was mediated, at least in part, by activating hippo signaling and upregulating miR-27b, which inhibits the expression of genes involved in all critical steps of lipid droplet biogenesis in hMSCs. On the basis of these results, quercetin could potentially be used as a dietary supplement to reduce adipogenesis by inhibiting lipid biogenesis in hMSCs. Although beyond the scope of the present study, we believe that additional experiments, such as an *in vivo* study using relevant animal models, should be carried out in the future to further clarify the effects of fisetin and quercetin on human adipogenesis, their underlying mechanisms, and the results of molecular docking analysis, before this knowledge can be applied in clinical application.

## Supplementary Material

Supplementary Figure S1 and Tables S1-S2

## Data Availability

All datasets in this article are included within the article and additional files.

## Abbreviations

DMEM: Dulbecco's Modified Eagle Medium
DMSO: Dimethyl sulfoxide
hMSCs: Human mesenchymal stromal cells
LATS kinase: Large tumor suppressor kinase
miR-27b: micro-RNA 27b
MSCs: Mesenchymal stromal cells
MTT: Methyl thiazol tetrazolium assay
STK: Serine/threonine protein kinase
YAP: Yes-associated protein
