# Supplementary material for: Effect of the polyphenol flavonoids fisetin and quercetin on the adipogenic differentiation of human mesenchymal stromal cells
Source: Biosci Rep. 2024 Oct 23;44(10):BSR20240623. doi: 10.1042/BSR20240623 (PMC11499385; doi:10.1042/BSR20240623)
Supplement: Supplementary Figure S1 and Tables S1-S2 [file BSR-2024-0623_supp.pdf]

Supplementary Fig 1

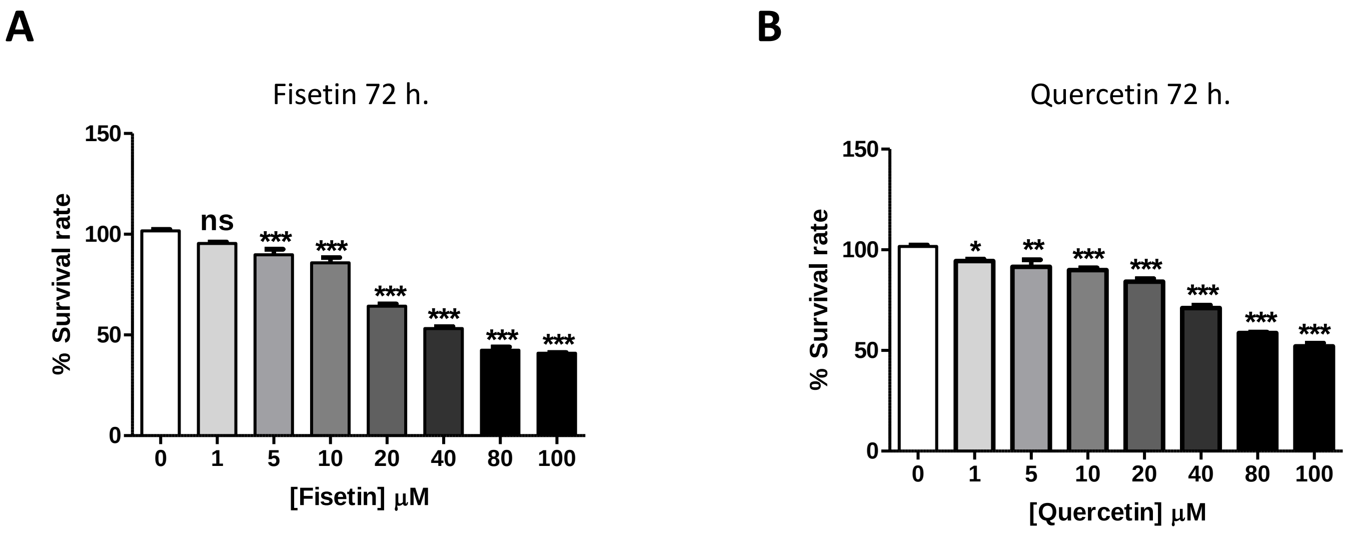

Supplementary Figure 1: MTT assay showing the viability of hMSCs after exposure to different concentrations of fisetin (A) and quercetin (B) for 72 h.

Full western blot membrane associated to figure 5A

Associated to figure 5A

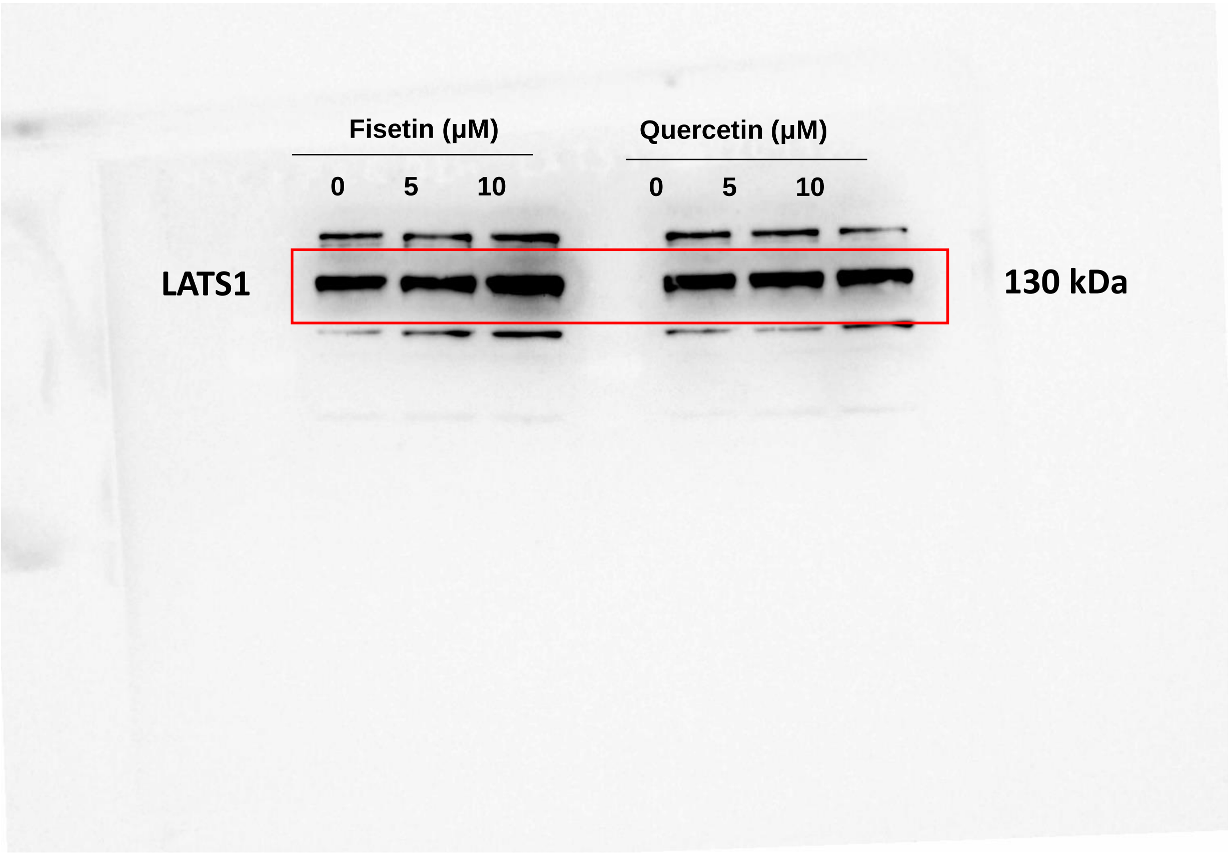

Associated to figure 5A

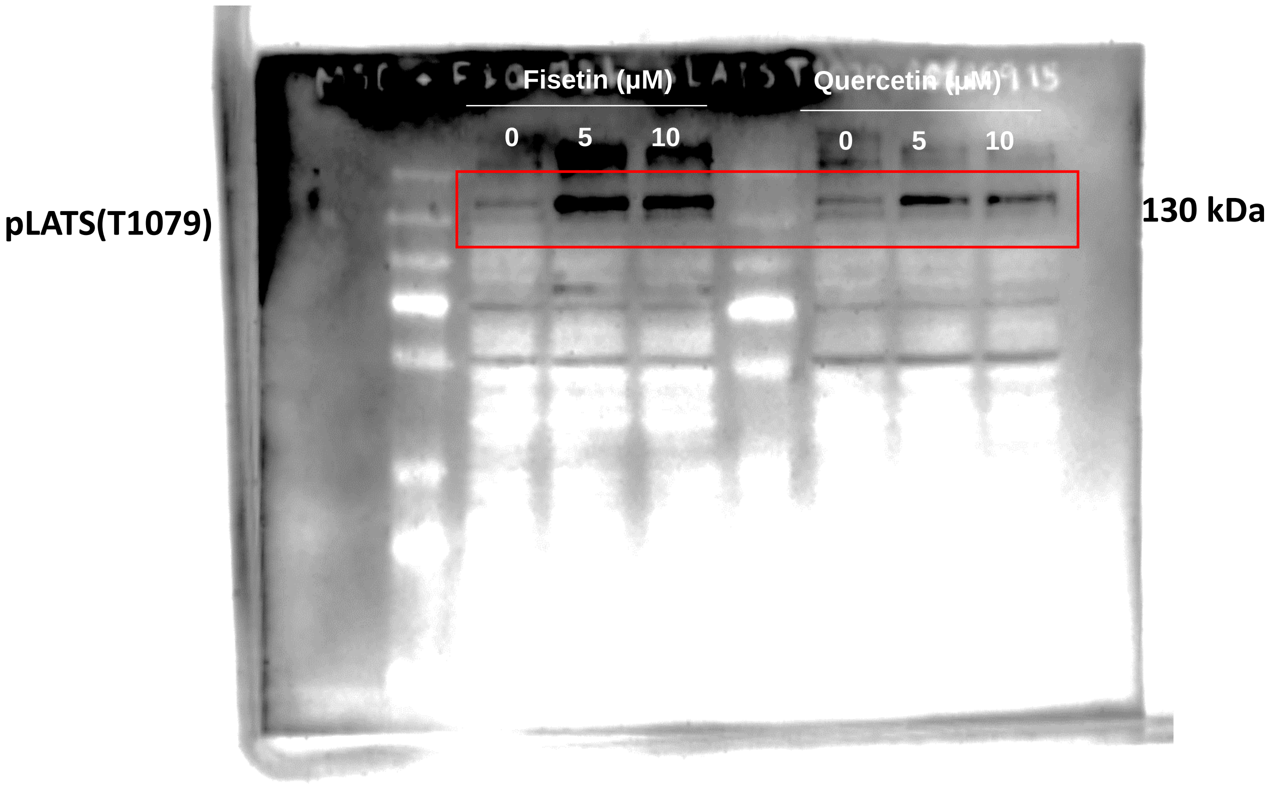

Associated to figure 5A

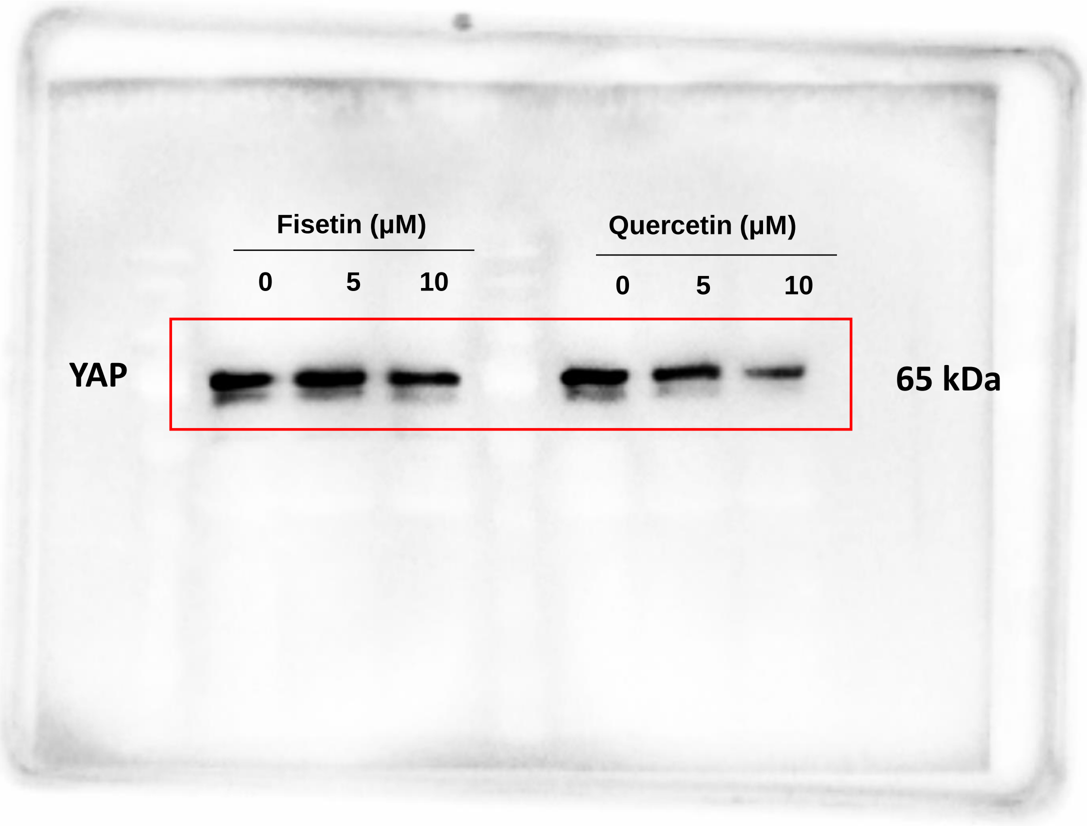

Associated to figure 5A

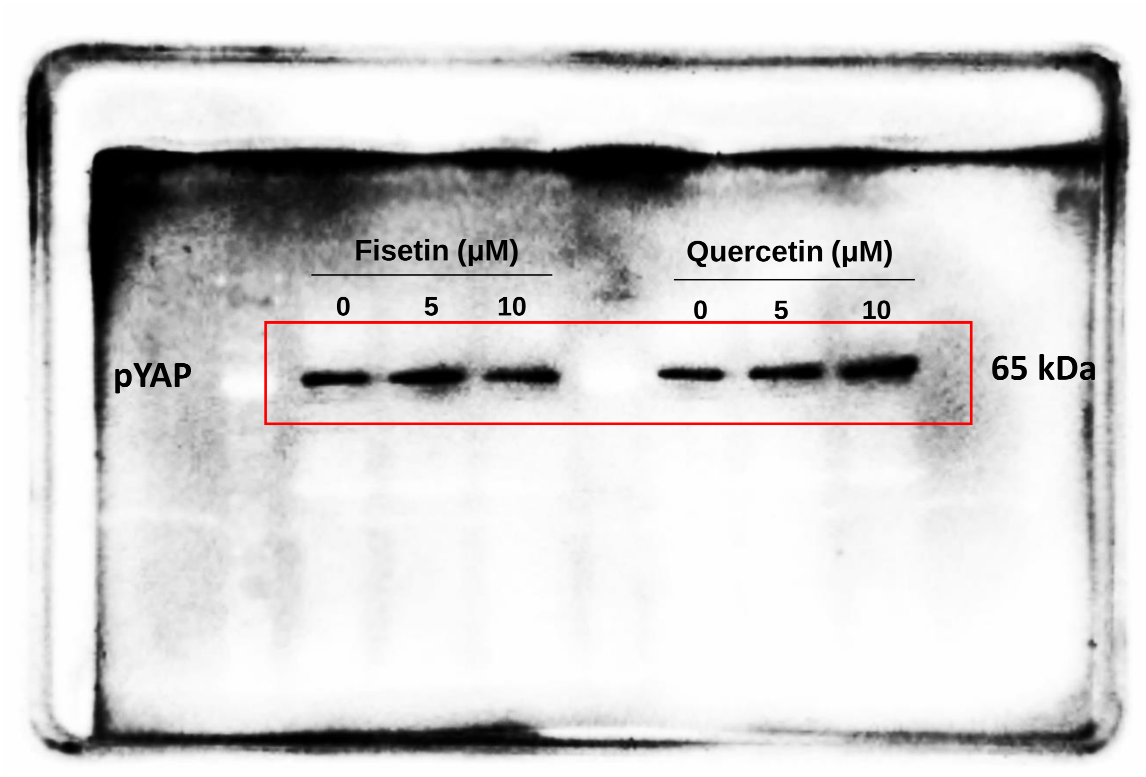

Associated to figure 5A

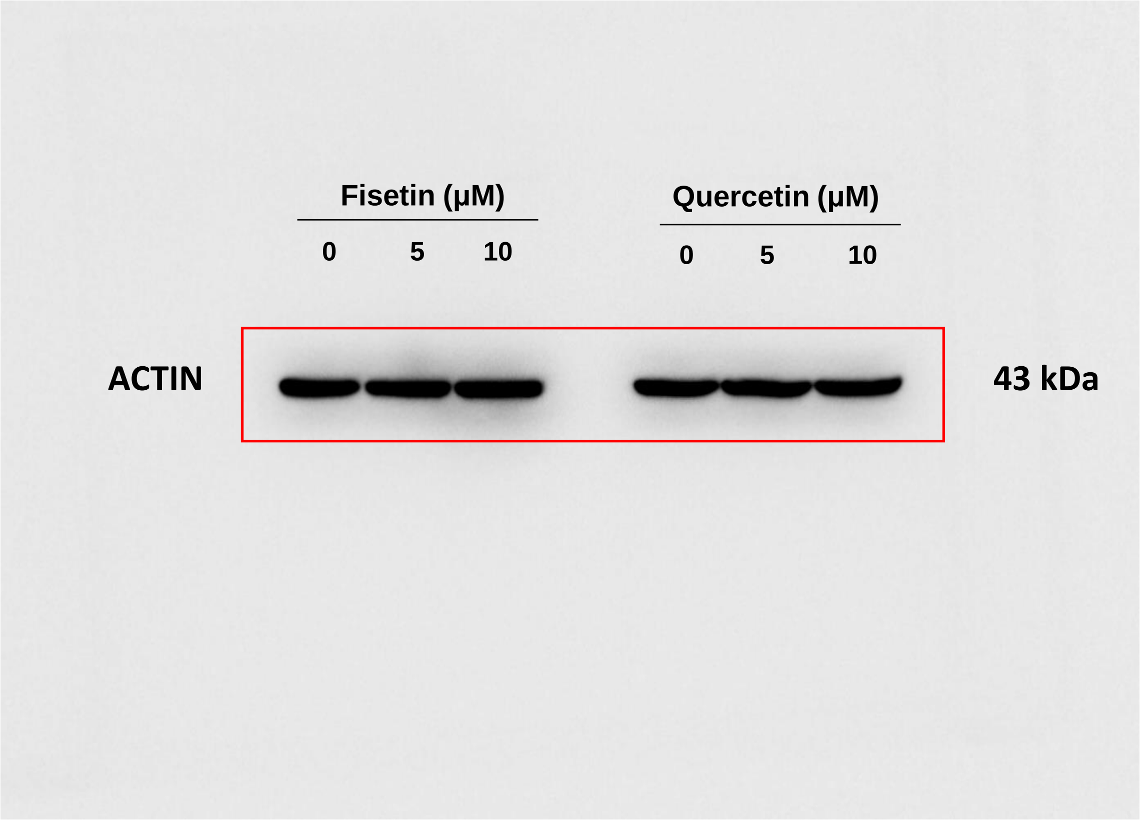

Supplementary Table 1. List of primers used for studying adipogenesis

Gene	Marker for	Forward primer	Reward primer
ADIPO-NECTIN	adipogenic	5'-TATCCCCAACATGCCCATTCG-3'	5'-TGGTAGGCAAAGTAGTACAGCC-3'
PPARG	adipogenic	5'-CCTATTGACCCAGAAAGCGATT-3'	5'-CATTACGGAGAGATCCACGGA-3'
GPAT2	Lipid forming	5'-GGCTGACGGAGGAGATACTG-3'	5'-AGTTGTGCCAGGTGTGTGAG-3'
LPIN1	Lipid forming	5'-AGAAGAACCCAGAAATGCTTTG-3'	5'-CTCTTTCATCTTGTGTGGAGAAG-3'
LPIN2	Lipid forming	5'-CCTCTCCTCAGACCAGATCG-3'	5'-GGAGAATCTGTCCCAAAGCA-3'
FITM2	Lipid forming	5'-GTACATCTGCACCTCCATCTTC-3'	5'-CTTGCTCTGGTGTTCCTTTCTG-3'
FITM2	lipid droplet budding	5'-GTACATCTGCACCTCCATCTTC-3'	5'-CTTGCTCTGGTGTTCCTTTCTG-3'
BSCL2	lipid droplet budding	5'-TTCTACTACAGGACCGACTG-3'	5'-CAGCTCAAGCTCTAAGGTAAC-3'
PLIN1	lipid droplet fusion	5'-AAGTTGAAGCTTGAGGAGCGAGG-3'	5'-GCTCGCGATGGGAACGCTGA-3'
CIDEC/FDP27	lipid droplet fusion	5'-GAGGTCCAACGCAGTCCA GCTG-3'	5'-GTACGCACTGACACATGCCTGGAG-3'
GAPDH	Glyceraldehyde 3-phosphate dehydrogenase	5'-GTCAACGGATTTGGTCGTATTG-3'	5'-CATGGGTGGAATCATATTGGAA-3'

Supplementary Table 2: List of TaqMan probe for miRNA study

Cat no.	Probe information
4427975	TaqMAn MicroRNA assays,50RT/150PCR (has-miR21-5P)
4427975	TaqMAn MicroRNA assays,50RT/150PCR (has-miR27b-3P)
4427975	TaqMAn MicroRNA assays,50RT/150PCR (has-miR29a-3P)
4427975	TaqMAn MicroRNA assays,50RT/150PCR (has-let-7b)
4427975	TaqMAn MicroRNA assays,50RT/150PCR (U6)
